# Feasibility of post-stroke hand rehabilitation supported by a soft robotic hand orthosis in-clinic and at-home

**DOI:** 10.1186/s12984-025-01717-6

**Published:** 2025-08-21

**Authors:** Natalie Tanczak, Tegan Kate Plunkett, Sijie Lin, Lorena Kuenzler, Megan Lau, Wee Keong Christopher Kuah, Chwee Yin Ng, Roger Gassert, Karen Chua, Olivier Lambercy

**Affiliations:** 1 Future Health Technologies, Singapore-ETH Centre, Campus for Research Excellence and Technological Enterprise (CREATE), Singapore, Singapore; 2 Rehabilitation Engineering Laboratory (RELab), Department of Health Sciences and Technology (D-HEST), ETH Zurich, Zurich, Switzerland; 3 Institute of Rehabilitation Excellence (IREx), Tan Tock Seng Hospital Rehabilitation Centre, Singapore, Singapore

**Keywords:** Robotic hand orthosis, Wearable robots, Stroke rehabilitation, Feasibility, Functional training, Neurorehabilitation, Upper limb

## Abstract

****Background**:**

Stroke is a leading cause of adult disability in the world. Upper limb impairments are common post-stroke, with nearly half of those initially affected continuing to live with long-term functional limitations, impacting their independence and quality of life. Task-specific, intensive therapy has been shown to promote recovery; however, achieving this dose is resource-intensive and logistically challenging. Robotic hand orthoses (RHOs) are a promising approach to support functional rehabilitation regardless of location, thus providing high-dose therapy with minimal additional burden on the clinics.

****Methods**:**

We evaluated the use of the RELab tenoexo 2.0, a soft RHO, in a two-phase feasibility study supporting functional hand training in persons after chronic stroke. Participants (n = 8) first used the device to complete nine training sessions supervised by occupational therapists over 3 weeks in-clinic, then brought the device home to continue the training to complete 2 weeks of unsupervised training. Feasibility was assessed with therapy dose (repetitions and time) and adherence to the suggested at-home rehabilitation program. Functional improvements were tracked using clinical assessments across time points. Finally, usability evaluations provided insights into users’ perceptions of the device.

****Results**:**

During the in-clinic phase, participants completed an average of 809 ± 317 RHO supported repetitions over 521 ± 130 min. At home, this increased to 1293 ± 948 repetitions over 486 ± 125 min across an average of 11.75 ± 5.4 sessions. Across the whole intervention, participant’s mean Action Research Arm Test score increased by 5.0 ± 4.4, whereas the Fugl-Meyer Assessment Upper Extremity score increased by 6.0 ± 2.5. These improvements were retained after one month. The usability was rated as good, with a mean System Usability Scale rating of 72.5, and a mean Quebec User Evaluation of Satisfaction with Assistive Technology 2.0 score of 3.94/5.0.

****Conclusion**:**

This study shows that the RHO can serve as a viable rehabilitation tool for functional hand training after chronic stroke across the continuum of care. High-dose training, both in-clinic and at home, demonstrated the feasibility of the device and intervention, with meaningful clinical improvements highlighting its therapeutic potential as a training strategy. High adherence rates and positive usability indicate strong user acceptance.

****Trial registration**:**

NCT06412237

## Introduction

There are about 12.2 million new strokes each year [1], making it the leading cause of adult disability in the world [2]. Among those with initial upper limb impairments, more than half will have persisting deficits even long post stroke [3]. These lasting upper limb impairments significantly hinder the ability to perform common activities of daily living (ADL). As a result, this negatively affects individuals’ quality of life (QOL) due to the reduction of abilities, independence and societal participation [4–6]. Functionally relevant, task-specific and intensive therapy can help regain some of these lost functions [7]. However, delivering the required high level of dose is difficult without increasing the required resources and associated costs.

Wearable robotics, specifically robotic hand orthoses (RHO), can complement conventional rehabilitation by helping to restore lost movement patterns and supporting task-specific training both in the clinic and at home while only adding minimal burden on the already limited resources in the clinics. Especially at home, compact, portable, and easy-to-use active RHOs could serve as rehabilitative tools that facilitate the delivery of higher therapy doses independent of time and place. An example of such a RHO is the RELab tenoexo, a soft robotic device based on a spring-blade mechanism offering a lightweight, tailorable, and fully wearable design [8]. The device’s benefits and usability have been evaluated in numerous studies [8–13], investigating its usage as either a rehabilitative or an assistive tool, both in spinal cord injury (SCI) and children with neurological hand impairments, and in different settings. Other studies [14–16] have also shown that different types of soft RHOs can prove helpful in supporting hand rehabilitation with favourable usability and clinical outcomes [17]. However, research on the feasibility of prolonged use of RHOs to support functional upper limb therapy in chronic stroke along the continuum of care remains limited. Specifically, it remains unclear how this population will perceive and adhere to the usage of a wearable, active RHO starting from in-clinic to at-home self-initiated training.

This work presents the results of a feasibility study in which an improved version of the RELab tenoexo was used to support occupational therapy for hand training in persons after stroke, first in-clinic and then transitioning to home. The primary outcome of this work was the achieved training dose, defined by the number of supported hand movements, and the adherence to the suggested rehabilitation protocol. Secondary outcomes included changes in participants’ arm impairment and functional ability, assessed through a series of clinical evaluations. Finally, the overall system usability was evaluated at multiple time points throughout. The key contribution of this work lies in its two-phase transitional training design, allowing for a comparison of device usage under varying levels of supervision and in different settings. We hypothesized that users could use the device frequently and consistently at home, achieving a dose comparable to in-clinic use. This sustained dose is expected to result in measurable and clinically meaningful upper limb functional improvements that are maintained over time. This work is important as it evaluates the suitability of a soft RHO for delivering functionally relevant therapy across the continuum of care and presents initial evidence of its clinical benefit for individuals with chronic stroke.

## Methods

### Soft robotic hand orthosis—RELab tenoexo 2.0

The RELab tenoexo 2.0 is a soft RHO that supports grasping in individuals with sensorimotor hand impairments. It comprises two parts: a hand module (270 g) and a backpack (411 g). The hand module mechanically supports grasping by helping to flex and extend the fingers using a sliding spring mechanism. An opposable thumb, that can be manually adjusted with a slider, expands the number of possible grasp types [8]. The softness of the RHO allows it to adapt to the natural contour of the hand, offering a high level of comfort and safety. This is particularly valuable for independent use at home, where ease of use and intuitiveness are essential for high use.

In this new design iteration of the RELab tenoexo 2.0, the design of the finger and actuation system were significantly reworked to improve the simplicity and robustness of the system. Notably, the actuation units are now enclosed within the hand module, as opposed to previously being in the backpack. The backpack now houses the electronics and battery required to operate the device. Both are connected via a USB-C cable to transmit trigger information. The RELab tenoexo 2.0 can support multiple different types of intention detection strategies depending on user preference [18]. Due to its reliability and robustness, in this study, a push button was used to trigger the device. The backpack automatically logs relevant device information, such as when the device is turned on, as well as time-stamping each trigger (indicating “open" or “close" trigger).

In past work, users achieved higher scores on clinical assessments when using tailored hand modules [12]. Therefore, in this study, custom hand modules were designed for each participant. Leading up to the study, a photo of each participant’s hand was taken, and measurements were extracted using a Computer Aided Design (CAD) software (Solidworks 2024, Dassault Systèmes, USA). These measurements were used to tailor each module to the participant’s finger length and palm width. A tailoring algorithm was used to generate the corresponding CAD files automatically. The respective springs, fingers, and palm plates were then manufactured accordingly. The rest of the hand module (including the actuation unit) and the backpack are standardized and reusable. Creating and exporting the required CAD files takes a matter of minutes. All ordered components can be received within a few days. Adding the custom components to the standardized parts can be done in under 30 min.

### Recruitment

This study was a feasibility trial for safety, usability, and clinical outcomes. It took place in the outpatient rehabilitation clinic of Tan Tock Seng Hospital (TTSH), National Healthcare Group, Singapore, from November 2023 to January 2025. Study inclusion criteria were individuals aged 21–80 years, with confirmed stroke and hemiplegia of at least >6 months, Montreal Cognitive Assessment (MoCA) score of $\ge$ 22/30 with the presence of a carer or next of kin who was able to help with home-based training, if required. Individuals had to have some remaining proximal function (shoulder abduction Medical Research Council (MRC) motor power >2/5 and elbow extension >2/5) and spasticity clinically graded by the Modified Ashworth Scale (MAS) $\le$ 2.

Before enrollment or study interventions, institutional review board ethics approval were obtained from the National Healthcare Group (NHG DSRB 2023/00448) and the ETH Zurich Ethics Commission (EK-2023-N-168-A). The study was registered with www.clinicaltrials.gov (NCT06412237). All research was conducted in accordance with the Declaration of Helsinki. All participants provided written informed consent before enrollment.

### Study protocol

**Fig. 1:**
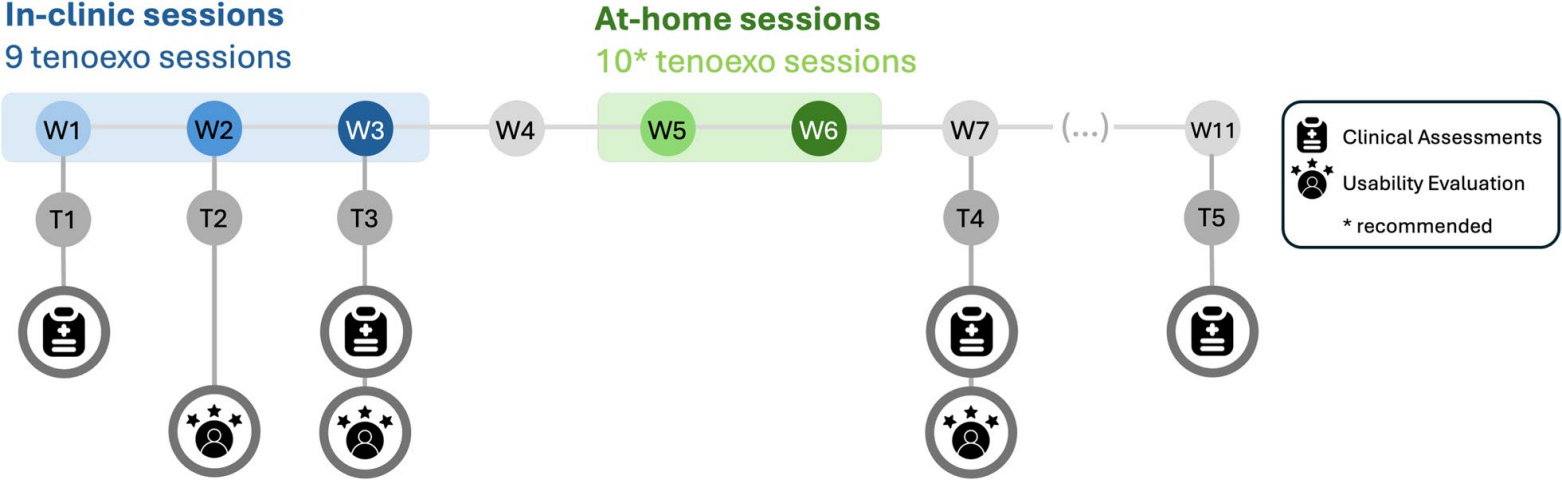
Study schedule. In-clinic training took place from W1–W3, and at-home training is from W5–W6. Clinical assessments were conducted in W1, T1 (baseline), W3, T3 (after in-clinic training), W7, T4 (after at-home training) and W11, T5 (retention). Usability evaluations were conducted at T2, T3 and T4

Each participant completed an 11-week protocol, which included baseline measurements and a follow-up assessment one month after the intervention to assess retention. During the study period, participants did not participate in any other structured therapy programs. A detailed schedule is provided in Fig. 1. The study was carried out in two phases, initially in the clinic (blue; W1–3), followed by a home-based phase where participants used the device independently (green; W5–6). About a month before the start of the intervention (T1), tailored devices were built for each participant.

**Fig. 2:**
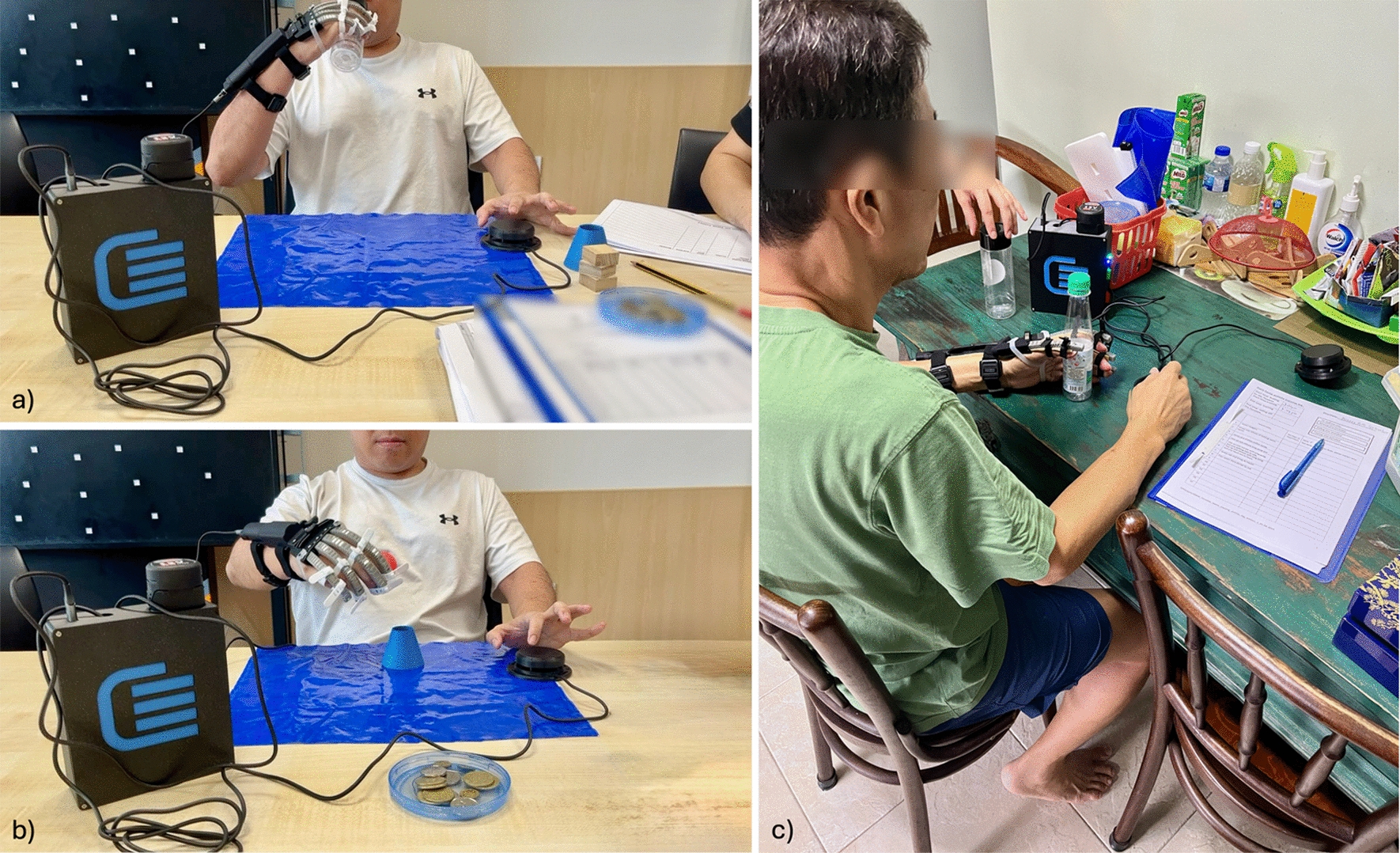
*Left* participant using the RELab tenoexo 2.0 during the in-clinic phase. Sample tasks include **a** drinking from a bottle and **b** reach-and-place task with ping-pong balls. *Right* participant during an at-home training session

The in-clinic phase (W1–3) comprised nine RELab tenoexo 2.0 supported training sessions. During this phase, trained occupational therapists (OTs) conducted functional training sessions where participants were asked to grasp and manipulate day-to-day objects (Fig. 2). All participants performed a series of standardised tasks (e.g., grasping various diameter bottles and ping pong balls), while additional objects and variations in task difficulty level (e.g., moving a bottle in front versus placing it on a shelf) challenged participants and kept them motivated and engaged during the session. If participants had specific tasks they wished to train, these were integrated into the sessions to personalise therapeutic goals. Throughout the sessions, OTs monitored movement patterns and minimised the use of compensatory strategies.

In W5 and W6, participants had the device at home and were instructed to perform at least ten, one-hour RELab tenoexo 2.0-supported sessions over the 14 days. These sessions did not have to be continuous and could be split into shorter durations (e.g., two 30-minute sessions). Each participant was given the same objects used during the in-clinic phase, along with a logging sheet to record usage and repetitions, and a list of suggested tasks that they could perform at home with the device. These tasks were the same as the ones performed during the in-clinic phase. Participants were informed that they had full autonomy in using the device and were permitted to modify, add, or remove tasks at their discretion. The research team did not monitor or intervene if the participants did not comply with the usage suggestion (i.e., to allow for an unbiased evaluation of adherence). Study team members were available on demand to support with any questions, troubleshooting, or repairs.

Clinical assessments were conducted at multiple time points to track functional improvements throughout the different phases of the trial. On the days of the first and last therapy sessions (T1 and T3), clinical assessments measured the baseline and post-clinic training ability levels, respectively. After the home phase, participants returned to the clinic to measure their post-intervention changes (T4). A final clinical assessment was conducted one month later (T5) to measure retention. All clinical assessments were performed by OT members of the research team. Standardisation of outcome measures was completed within the OT team to minimise variability in scoring.

Usability evaluations were conducted at three time points to understand how participants’ perceptions of the device and intervention changed throughout the different phases. These evaluations took place after the midpoint and final in-clinic sessions (T2, T3) as well as after the home phase (T4).

### Outcome measures

#### Feasibility

The achieved dose was calculated from the logged values from the RELab tenoexo 2.0 backpack. Once the backpack unit is turned on, a log file is initiated, continuously logging and saving data until the device is turned off. This log includes the commands sent to the hand module (either “open" or “close") and the corresponding timestamp. Each repetition is defined as a full grasp cycle, where the hand first closes and then opens while manipulating an object. Therefore, each reported repetition corresponds to two hand movements. To measure active training time, the length of a session is defined as the elapsed time between the first and last trigger within a single session. To only include active training time, during data analysis, significant breaks (lasting more than 5 min between consecutive triggers) were flagged and excluded from the calculation. This ensures that only continuous and active training time is reported.

The feasibility of using the device was measured through device usage, including the total time used, the total number of grasps, and the number of independent sessions performed at home. To facilitate comparison across settings, the data was normalised to grasps per hour. An adherence rate was also calculated to measure participants’ adherence to the suggested usage at home. The following equation was used to calculate the adherence rate based on both the total training time and the number of completed sessions. This was later compared to the recommended dose of ten, one-hour sessions.

$$Adherence \, Rate = \left( \frac{Actual\, Dose}{Expected\, Dose}\right) *100 $$

The safety of the system was measured by routinely asking participants whether they experienced any discomfort, pain, or adverse effects related to the device. These assessments were conducted by study staff members. Finally, the number of technical interventions required during the study period was recorded as an indicator of the system’s robustness.

#### Clinical assessments

A battery of clinical assessments was used to evaluate upper limb functional ability and impairment level throughout the intervention, as well as QOL. The Action Research Arm Test (ARAT) [19] was used to assess upper limb functional capacity. The maximum possible score is 57, and the Minimal Clinically Important Difference (MCID) for chronic stroke is 5.7 points [20]. The Fugl-Meyer Assessment for Upper Extremity (FMA-UE) is a subset of the full Fugl-Meyer assessment, specifically focusing on the sensorimotor function of the upper extremities in hemiplegic persons after stroke [21]. A total of 66 points is possible, with the MCID for chronic, moderate to mildly impaired individuals ranging from 4.25 to 7.25 points. In this work, we use an MCID of 5.75, the mean of the recommended improvement range [22, 23]. The Modified Ashworth Scale (MAS) was used as a validated clinical assessment to measure muscle tone [24, 25].

Two QOL metrics were used to evaluate the impact of the intervention on participants’ self-perceived well-being. The first was the EQ-5D-5L, a standardized instrument for measuring health status across five dimensions: Mobility, Self-Care, Usual Activities, Pain/Discomfort, and Anxiety/Depression [26]. For the analysis, the index score was computed, and the population norms for Singapore were used [27]. The second was the Stroke-Specific Quality of Life (SS-QOL), a validated tool designed to assess the impact of stroke on an individual’s QOL [28]. QOL scores were collected at three time points: baseline (T1), immediately after the intervention (T4), and at a one-month follow-up (T5).

#### Usability of the device

Two standardized and validated usability metrics were used to assess the acceptability of the device. The first was the System Usability Scale (SUS), a widely used and validated questionnaire for evaluating perceived usability [29]. SUS scores between 70 and 84 out of 100 are generally considered indicative of good usability [30]. Additionally, the Quebec User Evaluation of Satisfaction with Assistive Technology (QUEST 2.0) was employed to capture users’ satisfaction with both the assistive device and the related services [31].

### Data analysis and statistics

Descriptive statistics were computed using RStudio. Friedman tests were used to assess differences across time points, followed by pairwise Wilcoxon signed-rank post-hoc tests when significant effects were observed. Clinically meaningful changes in ARAT and FMA-UE scores were evaluated using established MCID thresholds. Data visualization was conducted in MATLAB (R2024b, The MathWorks, Natick, USA).

## Results

### Participants

Eleven participants were screened, and eight were enrolled (n = 1 not eligible due to an uncontrolled medical condition, n = 2 were unable to commit for the duration of the study). All eight recruited participants completed the entire protocol. Detailed participant demographics can be found in Table 1.

**Table 1:** Participant demographics

ID	Sex	Age	Time post stroke (m.)	Baseline FMA-UE	Baseline ARAT
S01	F	35	26	31	5
S02	F	52	19	50	42
S03	M	61	8	45	20
S04	F	69	33	40	26
S05	M	37	10	52	43
S06	M	59	26	31	11
S07	F	46	6	56	37
S08	M	48	6	51	35
Average	4F/4M	50.9	16.8	44.5	27.4
SD	–	11.8	10.6	9.6	14.3

Time post-stroke is reported in months from the date of recruitment

### Feasibility, achieved dose and safety

**Fig. 3:**
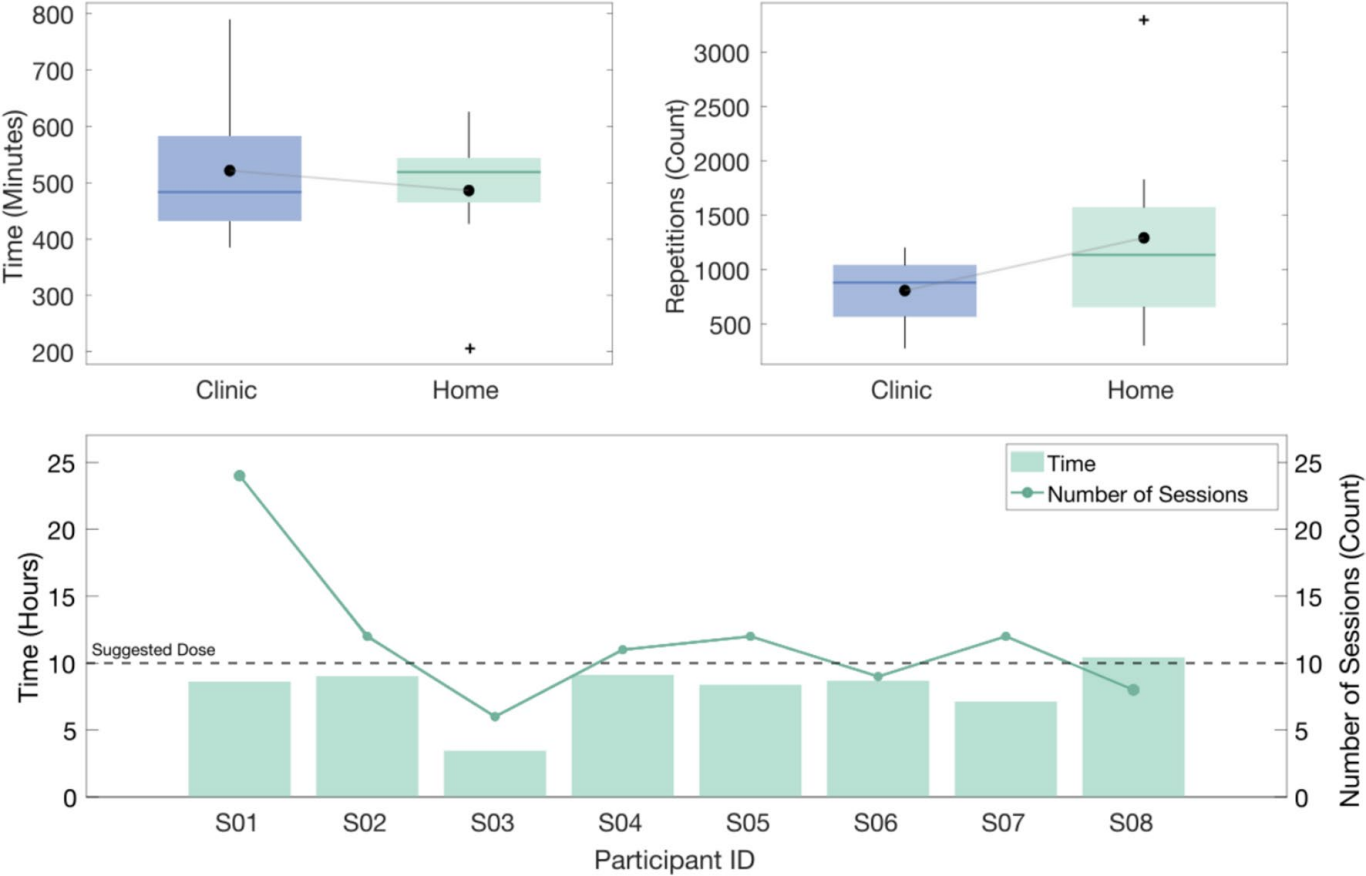
Overall device usage data. *Top left* time recorded of active usage in the clinic and at home. The mean is depicted with a connected *black dot*. *Top right* number of repetitions recorded in the clinic and at home. The mean is depicted with a connected *black dot*. *Bottom* Device usage at home. Comparison to suggested dose (*dashed line*) in terms of time (*left axis*) and number of sessions (*right axis*)

Throughout the intervention, both time of use and repetition data were collected from the backpack (Fig. 3). Across nine sessions in the clinic, participants performed an average of 809 (317) functionally relevant repetitions over 521 (130) min, reported as mean (SD). At home, this value increased to an average of 1293 (948) repetitions, over 486 (125) min across an average of 11.75 (5.4) sessions. In total, participants completed an average of 2102 repetitions over 1007 min.

At home, participants were advised to complete a total of 10 h of training with the device, as suggested by the study team. Participants were informed that they were permitted to use the device at their discretion, both in terms of manner and timing of use. Each participant’s adherence to the suggested usage can be seen in Fig. 3, where the raw active usage time of the device (excluding set-up time and breaks longer than 5 min) is presented. On average, participants achieved 81% of the suggested active usage time and 118% of the suggested number of sessions. Previous work has shown that in-clinic, caregiver-supported donning time for SCI patients averages approximately five min, with a maximum of nine min [11]. For independent use in a home setting, an average of 15 min was assumed for setup and wrap-up per one hour session. Under this assumption, the adherence rate to usage time increased to 108%. Across all participants, the timing and days on which the training occurred was analyzed. The most common training time was in the afternoon (39%), followed by evenings after 18.00 h (35%) and then mornings (26%). The most sessions occurred on a Monday (18%), followed by Thursday and Friday (15% each), Tuesday and Wednesday (14% each), and then finally Saturday (13%) and Sunday (11%).

When comparing normalized grasp data, in-clinic users performed 98.2 (46.9) grasps per hour, whereas at home, this increased to 150.8 (82.9). Six of the eight participants increased their hourly dose rate compared to in-clinic usage; however, no significance was found when comparing the two conditions (*p* = 0.08).

No pain or adverse events were reported throughout the study based on verbal inquiries raised during and at the end of each clinical session.

### Clinical scores

#### Clinical assessments

The ARAT and FMA-UE scores across all time points are summarized in Table 2. After the intervention (3 weeks in the clinic and 2 weeks at home), most participants showed improvements in both ARAT and FMA-UE. The ARAT scores increased by an average of 5.0 (4.4) points, reported as mean (SD). A Friedman test revealed a significant effect of time, and post-hoc pairwise comparison (Wilcoxon signed-rank) showed significant differences between T1–T4 and T1–T5. FMA-UE over the same period increased an average of 6.0 (2.5) points. A Friedman test again showed a significant effect of time, and the post-hoc Wilcoxon pairwise comparisons showed significance between T1–T3, T1–T4, and T1–T5. After a one-month retention period (T5), there were no significant changes in ARAT and FMA-UE scores (*p* = 0.999, *p* = 0.292, respectively) compared to after the intervention (T4). No notable differences were observed in MAS scores throughout the intervention (see Supplementary Material Table 1).

**Table 2:** ARAT and FMA-UE clinical outcomes over four time points

ID	ARAT					FMA-UE				
	T1	T3	T4	T1 to T4	T5	T1	T3	T4	T1 to T4	T5
S01	5	6	10	+5	11	31	33	32	+1	28
S02	42	41	40	-2	42	50	57	59	+9*	58
S03	20	24	22	+2	29	45	49	53	+8*	56
S04	26	27	35	+9*	34	40	49	46	+6*	46
S05	43	39	50	+7*	45	52	55	59	+7*	58
S06	11	16	16	+5	13	31	39	38	+7*	36
S07	37	40	49	+12*	51	56	60	62	+6*	62
S08	35	36	37	+2	35	51	53	55	+4	53
Average	27.4	28.6	32.4	+5.0	32.5	44.5	49.4	50.5	+6.0*	49.6
SD	14.3	12.8	14.9	4.4	14.4	9.6	9.2	10.8	2.5	12.0

Three of these occurred during the intervention (T1, T3, T4), with one (T5) occurring after as the retention measurement. Clinical meaningfulness as defined by each respective MCID is marked with *

#### Health-related quality of life

No statistically significant differences were observed in the health-related QOL measures (SS-QOL (*p* = 0.053) or EQ-5D-5 L (*p* = 0.651)) across the intervention period (see Supplementary Material Table 2).

### Usability scores

The summarised usability outcomes are presented in Table 3. The overall mean SUS score was 72.5, resulting in a good rating [30]. The mean QUEST 2.0 score was 3.94 (0.42). Within the QUEST 2.0 scores, the mean score for the assistive device was 3.66 (0.47), and the services delivered was 4.50 (0.59). The lowest subscores were questions 3 (3.33/5) and 5 (3.13/5), which refer to the ease in adjusting the device (e.g., fixing and fastening) as well as the durability (endurance, resistance to wear). The highest ratings were for questions 4 (4.25/5) and 6 (3.94/5), which referred to the safety and security of the device, as well as its ease of use. No significant differences were seen over time for both SUS (p = 0.497) and QUEST 2.0 (*p* = 0.166).

### Required interventions

No serious adverse events were reported during the study. Minor technical support was required, primarily due to the durability of the finger material. The RHO fingers sometimes broke unpredictably but were easily replaceable without the need to doff the device. Both the engineer and OTs were trained to perform such replacements. During the at-home phase, study personnel were available on demand to make home visits as needed, with the number of visits ranging from zero to five per participant. Midway through the study, switching to a more robust finger material resolved this issue. Other technical adjustments included reinforcing weak solder connections on the USB-C connector and replacing palm plates that developed minor cracks at their connection points. Most repairs were completed on-site; however, on two occasions, the use of a soldering iron was required, necessitating off-site repair.

**Table 3:** SUS and QUEST 2.0 results from three time points (during in-clinic training, after in-clinic training, and after home training)

	SUS		QUEST 2.0	
	Average	Range	Average	Range
T2—Mid in-clinic training	72.19	[52.5, 82.5]	3.78	[3.17, 4.5]
T3—Post in-clinic training	75.63	[55, 92.5]	4.03	[3.42, 4.75]
T4—Post home training	69.69	[45, 82.5]	4.02	[3.42, 4.08]
Overall average	72.5	[45, 92.5]	3.94	[3.17, 4.75]

Results from all participants were averaged, and the range is provided

## Discussion

In this work, we presented and evaluated the feasibility of using a soft RHO to support functional hand training for persons after chronic stroke. We implemented a two-phase, transitional training protocol to facilitate this. By first evaluating the achieved dose, we demonstrated that the device can be used under full supervision in-clinic as well as through self-guided usage at home. Furthermore, positive clinical and usability outcomes confirmed the intervention’s feasibility and participants’ favorable experience with the device.

### Using soft robotics for rehabilitation along the continuum of care

The primary outcome of this study was the achieved training dose and adherence to the suggested usage protocol. Using RELab tenoexo 2.0, participants achieved high training doses, completing up to an average of 150.8 repetitions per hour of training, regardless of location. This quantity is nearly five times greater than what was reported in conventional task-specific functional training, which sees an average of 32 completed functional repetitions per session (e.g. reaching for a cone) [32]. Moreover, this dose could be achieved even in the absence of a therapist at home, demonstrating that this approach can deliver the required high dose of therapy while alleviating the burden on the already strained clinical resources.

Our results compare favourably to other similar studies that reported repetition-based dose. For example, Yurkewich et al. achieved a supported dose of 56 grasps per hour in the clinic, but no data was available for the home phase [16]. Proietti et al. achieved 319 full repetitions (open and close) per hour. Their work, however, did include app-based training, which was likely the enabler for achieving such a high dose [14]. Regarding time-based dose, our participants performed an average of 486 min of therapy over 14 days, or 34.7 min per day. This exceeds the results of the therapy group in Radder et al., who used the device for 21 min per day over 4 weeks [33] and the 17.8 min per day again over 4 weeks reported by Proietti et al. [14].

The two-phase structure of the study, comprising in-clinic and at-home training, also provided insights into usage patterns. The in-clinic phase served both as supervised therapy and as a structured familiarisation period. During this time, participants learned about the device’s application (what tasks it can support) and the specifics of how it works (donning/doffing and turning on the device). This transitional phase likely contributed to the high confidence, adherence, and independent usage observed at home. Such approaches have previously been shown to ease the transition to self-directed rehabilitation [34, 35].

Contrary to our expectations, device usage did not decline once participants moved to self-administered, independent usage at home. Although the total therapy time decreased at home, we saw a 1.5-fold increase in grasps per hour compared to the in-clinic sessions. These results could be explained by a range of reasons: high self-motivation, the benefit of self-paced usage, fewer distractions at home (e.g., socializing with clinicians), less emphasis on perfect form (as dictated by therapists in the clinic), and greater comfort and confidence in their familiar home environment. Further, training sessions frequently occurred outside of typical clinic hours, with 35% being held in the evenings, and 24% being held on weekends. This underscores the flexibility that at-home training provides, allowing participants to engage at their own convenience. Ultimately, these findings highlight how soft wearable technology can meaningfully complement conventional therapy by enabling high-dose, self-directed rehabilitation outside the clinical setting.

### Preliminary evidence of clinical benefits following RHO training

Throughout the intervention, we saw clinically meaningful improvements with several participants achieving MCID for both ARAT (3 out of 8) and FMA-UE (6 out of 8). Importantly, these gains were retained at the one-month follow-up. These outcomes are particularly noteworthy given that participants were in the chronic stage post-stroke and that our intervention focused solely on training the hand. This work adds to the growing evidence that functional, technology-supported hand training can benefit the overall upper limb function [36–38].

Our functional gains surpass those reported in other studies with comparable durations and intensities. For FMA-UE, we saw gains of +6.0 (2.5) over 5 weeks, compared to +3.9 (4.0) after 4 weeks [14], and +5.60 (2.61) after 6 weeks [39] in other work. For ARAT, participants improved by +5.0 (4.4) points over 4 weeks, significantly exceeding the +0.20 (2.17) reported after 6 weeks of training [39].

Beyond the presented clinical outcomes, a notable functional improvement observed in numerous participants was the newfound ability to voluntarily adduct and abduct the thumb. This new movement pattern is likely attributed to the unique design of the RELab tenoexo 2.0, where the manual slider of the thumb allows users to correctly position their thumb before grasping objects of different sizes. Repeated guided thumb movement during training may have helped to regain these functions. These results were evident in a sub-analysis of the ARAT results, which showed improvements in the *pinch* subscore, where three participants gained five points, with an overall average improvement of +2.13.

Despite these functional gains, no significant improvements were seen in the perceived QOL measurements. This may be due to the study’s short time frame and the limited time for functional gains to translate into perceived everyday benefits. However, these findings align with work that has shown an increase in capacity does not necessarily translate to performance [40]. Furthermore, the nature of the questionnaires, as seen in the SS-QOL example, reveals that many questions are unrelated to the intervention, including mood, personality, vision, and thinking. EQ-5D-5L similarly looks at mobility, pain, and anxiety/depression. This can potentially dilute any observable impact of the intervention.

### Usability and acceptance

Following the in-clinic training, most participants were able to don the device independently at home. Minor assistance was required for some participants (e.g., S01, S02, S04, S06) to help extend their fingers, as they have increased muscle tone in their finger flexor muscles. Nonetheless, the strapping process itself was well-tolerated by all participants. Adjustments made during the in-clinic phase also helped to adapt each device to each participant’s preferences and physical abilities. This underlines the importance of an in-clinic familiarisation phase to optimise how neurological patients use new rehabilitation technology before being given it to take home [41]. In future studies, the necessity of having a carer at home to help with training could be removed as an inclusion criterion and instead be judged on a case-by-case basis.

Participants provided positive feedback regarding the study design, specifically about the in-clinic tasks and exercises performed at home. Despite the high repetition rate and repeatability of tasks between sessions, participants valued the challenge and were motivated by their visible progress. Participants reported that tasks became either easier or faster with time, reinforcing their motivation to continue the therapy.

The increased usage at home was supported by participants’ feedback. The at-home phase was generally well-received, with participants praising the freedom and flexibility that at-home therapy gave them. Some participants found it challenging to multitask during home sessions, where they were asked to record the number of repetitions of specific tasks on the logging sheet. For these participants, this was reflected in lower usage of the device at home (e.g., S03). In hindsight, had this feedback been conveyed during the intervention, participants could have been advised to concentrate solely on the exercises, which may have contributed to an increased overall training dose.

According to the SUS scores, the usability of the device was rated as good according to the defined scale by Bangor et al. [30], with a mean score of 72.5. This score is comparable to other trials [33], yet still leaves room for improvement for higher scores [14, 16] to ensure a higher probability of acceptance. QUEST 2.0 also allowed us to understand the influence of the therapists, support engineer, and the overall services provided throughout the intervention. While the mean total score was high (3.94/5), if one looks at the device versus services subscores, there were considerable differences in the respective average scores (3.66/5 and 4.5/5). The lower scores can be attributed to reliability issues associated with the breakage of the RHO fingers, a problem that was addressed toward the end of the study and is expected to improve in subsequent design iterations. Although the results demonstrate feasibility within a research context, further refinements, particularly to the donning and attachment methods, are necessary to ensure successful clinical translation and scalability.

### Limitations and future work

This work faced limitations that may impact the results and their interpretation. Firstly, the OTs were not blinded and had previously treated some of the participants, which could have introduced potential selection bias. Additionally, technical challenges with the device caused interruptions in sessions when minor fixes were required. To address these challenges, another design sprint is required to further increase the technology readiness level of the RHO. Finally, insights into at-home use, such as set-up time or the amount of support provided by an external individual, were limited due to poor completion of the at-home log.

Extending the at-home period would allow for a better understanding of long-term adherence and suitability of the intervention. Future studies should also consider recruiting a larger and broader range of participants to have a better understanding of the device’s benefits. Finally, these studies should also carefully evaluate different models for implementing this type of therapy outside of a clinical study (e.g., access to technology, reimbursement models, etc.).

## Conclusions

This study positively demonstrates that the RELab tenoexo 2.0 can be used as a rehabilitation tool for functional hand training after stroke. Participants maintained a high level of training dose both in clinical settings and independently at home. High adherence rates and positive usability results indicate strong user acceptance. Finally, clinically meaningful and lasting improvements reinforce the hand exoskeleton’s feasibility as a training strategy. While the device and protocol were overall well-received, minor design adjustments are necessary to enhance the device for prolonged use, ensuring greater functionality and user satisfaction moving forward.

## Supplementary Information

Additional file 1.

## Data Availability

No datasets were generated or analysed during the current study.

## Abbreviations

RHO: Robotic hand orthosis
ADL: Activities of daily living
QOL: Quality of life
SCI: Spinal cord injury
CAD: Computer aided design
TTSH: Tan Tock Seng Hospital
MoCA: Montreal Cognitive Assessment
MRC: Medical Research Council
OT: Occupational therapist
ARAT: Action research arm test
MCID: Minimal clinically important difference
FMAUE: Fugl Meyer assesment for upper extremity
SSQOL: Stroke specific quality of life
SUS: System Usability Scale
QUEST 2.0: Quebec user evaluation of satisfaction with assistive technology
