# Supplementary material for: Feasibility of post-stroke hand rehabilitation supported by a soft robotic hand orthosis in-clinic and at-home
Source: J Neuroeng Rehabil. 2025 Aug 21;22:183. doi: 10.1186/s12984-025-01717-6 (PMC12369101; doi:10.1186/s12984-025-01717-6)
Supplement: Supplementary file 1 — Additional file 1. [file 12984_2025_1717_MOESM1_ESM.pdf]

Feasibility of post-stroke hand rehabilitation supported by a soft robotic hand orthosis in-clinic and at-home

Tanczak, N., Plunkett, T. K., Lin, S., Kuenzler, L., Lau, M., Keong, W., Kuah, C., Ng, C. Y., Gassert, R., Chua, K., & Lambercy, O.

Supplementary Material

Table 1: MAS scores before and after intervention for different muscle groups. Values represent mean (SD). The statistical significance of the changes was assessed using a paired t-test, with p-values indicated.

Muscle Group	Baseline	Post-Intervention	p-value
**Shoulder adductors**	0.13 (0.35)	0.25 (0.46)	0.60
**Biceps**	1.13 (0.23)	0.94 (0.42)	0.35
**Wrist flexors**	1.19 (0.65)	1.06 (0.56)	0.17
**Wrist extensors**	0.00 (0.00)	0.00 (0.00)	1.00
**Finger flexors**	1.00 (0.53)	0.94 (0.68)	0.68
**Finger extensors**	0.13 (0.35)	0.00 (0.00)	0.35

Table 2: Baseline scores (mean (SD)), Friedman test results, and Wilcoxon signed-rank post hoc comparisons for SS-QOL and EQ-5D-5L index scores across time points. P-values for pairwise comparisons are Bonferroni-adjusted.

Measure	Baseline	Friedman Test (χ^2, p)	Pairwise Comparisons (p)
**SS-QOL**	177.38 (24.49)	$\chi^2(2) = 5.87,\ p = 0.053$	T1 vs T4: $p = 0.699$<br>T1 vs T5: $p = 0.126$<br>T4 vs T5: $p = 0.453$
**EQ-5D-5L**	0.78 (0.18)	$\chi^2(2) = 0.86,\ p = 0.651$	T1 vs T4: $p = 1.000$<br>T1 vs T5: $p = 1.000$<br>T4 vs T5: $p = 1.000$
